# Self-Polarization of PVDF Film Triggered by Hydrophilic Treatment for Pyroelectric Sensor with Ultra-Low Piezoelectric Noise

**DOI:** 10.1186/s11671-019-2906-1

**Published:** 2019-02-28

**Authors:** Yuming Wu, Xiaosong Du, Ruoyao Gao, Jimeng Li, Weizhi Li, He Yu, Zhi Jiang, Zhidong Wang, Huiling Tai

**Affiliations:** 1 State Key Laboratory of Electronic Thin Films and Integrated Devices, School of Optoelectronic Science and Engineering, University of Electronic Science and Technology of China, Chengdu, 610054 China; 2 Department of Electrical Engineering and Information Systems, Graduate School of Engineering, University of Tokyo, Tokyo, Japan; 3 BOE Technology Group Co., Ltd., Chengdu, China

**Keywords:** Polyvinylidene fluoride, Pyroelectronic, Piezoelectronic, Flexible, Sensor

## Abstract

Polyvinylidene fluoride (PVDF) films possess multifunctional ability for piezo/pyro/ferroelectronic applications. One critical challenge of the traditional techniques is the complicated fabrication process for obtaining the poled films. In this work, the PVDF film is facilely prepared by the solution cast on hydrophilically treated substrates. The obtained PVDF films exhibit fairly good pyroelectricity comparable to those fabricated by thermal poling, indicating the film is self-polarized. This result is attributed to the hydrogen-bonding-induced orderly arrangement of the first sub-nanolayer at the bottom, which serves as a “seed layer” and triggered alignment of the rest of the film in a layer-by-layer approach. Additionally, to suppress the piezoelectric noise, a pyroelectric sensor with a novel bilayer structure is developed using the as-prepared PVDF film. Compared with the conventional monolayer sensor, the signal-to-noise ratio of the bilayer one is drastically improved to 38 dB from 18 dB. The above results provide great possibilities for achieving a high-performance wearable pyroelectric sensor with reduced cost and simple procedures.

**Electronic supplementary material:**

The online version of this article (10.1186/s11671-019-2906-1) contains supplementary material, which is available to authorized users.

## Introduction

Polyvinylidene fluoride (PVDF) and its copolymers [1–5] have become hot candidates for wearable electronics, multifunctional flexible sensors, and nano-generators in recent years due to their good piezoelectric and pyroelectric performance, flexibility, and ease of process [6–11]. However, it is still a great challenge to realize good pyroelectric function in PVDF. In conventional methods, two steps, i.e., stretching and thermal poling, are inevitable. The first step is to obtain a high β-phase content [12–16], and the second one is to further orient the dipole vectors in β phase normal to film surface [17–20]. The complicatedly obtained PVDF samples show disadvantages such as small active area, large amount of defects, low efficiency, and careful anti-electric shock proof [12, 13, 18–20]. Furthermore, due to the intrinsic piezoelectric nature of PVDF, traditional infrared sensors made from monolayer PVDF are fragile to environmental vibration noises, which greatly deteriorate the pyroelectric performance of the device.

Recently, various methods have been developed to realize self-polarized PVDF films without undergoing thermal poling, including casting [21–25], spin coating [26, 27], Langmuir-Blodgett (LB) deposition [28], electrospinning [29–35], and depositing onto aqueous salt solution [36]. In general, self-polarization of the PVDF films can be observed through the above techniques due to different mechanisms, such as the salt-assisted [21–25], hydrogen-bonding interaction [21–25, 27, 36], built-in field [26] or strong electric field [29, 35] during deposition, and stretching during coating [26, 28, 36]. Yet, most of these methods only focused on the piezoelectric performance of PVDF films and neglected its pyroelectric property. In addition, spin coating and LB techniques were only applicable for ultra-thin films [26, 28], while the casting method needed salt additive to achieve self-polarization [21–25], and the polarization mechanism of the electrospinning required further understanding [29–35]. When it turns to the issues of the sensor, selectively poling of ferroelectric ceramic-doped PVDF composites is a common method to decrease the effect of environmental vibration noise [37, 38]. These doped ceramics, e.g., lead zirconate titanate (PZT), have the same sign of pyroelectric coefficient (*p*) while the opposite sign of piezoelectric one (*d*_33_) as PVDF (or its copolymer). Thus, if two phases are polarized in parallel, the pyroelectric response will reinforce and the piezoelectric activity will partially cancel, which can reduce the vibration-induced electrical noise in pyroelectric sensors. However, the whole procedures are quite complicated; besides, after doping with ceramic, the dielectric properties of PVDF will be deteriorated, severely limiting the efficiency of this technique [39]. Therefore, it is still a great challenge to efficiently obtain a high-performance pyroelectric film as well as the sensor.

In this work, we develop a facile technique for the preparation of pyroelectric PVDF film by combining the conventional casting method with hydrophilic modification of the substrate. The results reveal that the as-prepared PVDF films simultaneously achieve a high β-phase content and a significant pyroelectric response. A polarization mechanism based on dipole-alignment-relay process is introduced to elucidate the above results. In addition, by using the prepared PVDF sample as the sensitive material, a bilayer structured flexible infrared sensor is proposed for achieving ultra-low piezoelectric noises in the device. This technique shows great potential to be applied in wearable infrared sensors or temperature sensors in harsh environment where large acoustic noises and/or mechanical vibrations exist.

## Methods

### Preparation of the PVDF Film and the Bilayer Pyroelectric Sensor

**Fig. 1:**
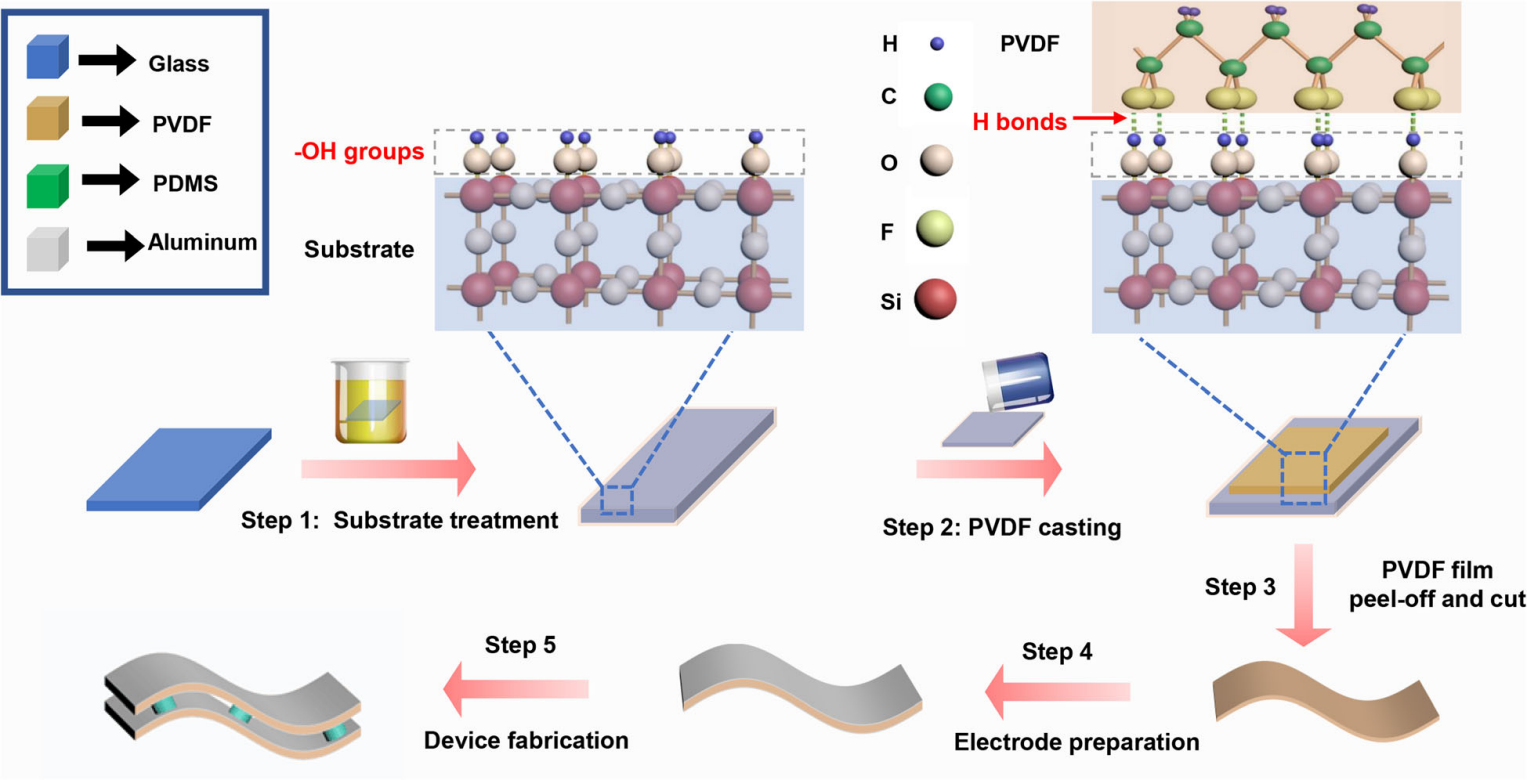
Illustration of PVDF film and device preparation process. Step 1, the glass substrate was soaked in piranha solution for 2–8 h. Step 2, well-stirred PVDF solution was cast on the substrate and dried at 80 °C for 10 h. Step 3, the PVDF film was peeled off from the substrate, and the edge was cut off to remove edge effect. Step 4, aluminum was evaporated onto both sides of the film as electrodes. Step 5, the bilayer device was fabricated by using PDMS pillars supported between the two layers as separators. Also indicated were schematics of the hydroxyl groups bonded on the surface of the glass substrate after treatment, hydrogen bonds formation after PVDF casting and orderly arrangement of the “ultra-thin layer” at the bottom of PVDF film

The preparation process of the PVDF film is shown in Fig. 1. Firstly, a piece of glass substrate was immersed in the piranha solution (mixture: H_2_SO_4_ (98% concentration, Kelong Chemical, China) and H_2_O_2_ (30% concentration, Kelong Chemical, China) with volume ratio of 7:3) for the hydrophilic treatment. The solution was placed in an incubator at 60 °C for a period of 2–8 h. A certain amount of PVDF powder (average M_w_ ~ 534,000, Sigma-Aldrich, USA) was blended with *N*-methylpyrrolidone (NMP) (99% purity, Kelong Chemical, China) solvent with a mass ratio of 10 wt%, which was then heated at 50 °C with magnetic stirring for 4 h until a completely uniform solution was achieved. This obtained solution was casted on the aforementioned treated substrate and kept at 80 °C for 10 h to remove the NMP solvent. To reduce edge effect in the films, the as-prepared PVDF film with an area of 10 mm × 10 mm was finally obtained by cutting the sample from the central area of the casted 50 mm × 50 mm film. For comparison, the PVDF sample was also fabricated on an untreated substrate and the thickness of all samples are 50 μm. Aluminum electrodes were evaporated on both sides of the samples for pyroelectric and piezoelectric performance measurement.

Holes with diameters of 1 mm throughout a 1-mm-thick acrylic plate (Xintao Plexiglass, China) were made by a high-power laser beam (type 4060, Ketai, China) and used as pillar models. Silicone elastomer (Sylgard 184, DOW CORING) was chosen as the pillar precursor material. The base and curing agents were mixed with a weight ratio of 10:1, which was then dropped into the holes. Polydimethylsiloxane (PDMS) pillars can be obtained after being cured at 60 °C for 10 h. The bilayer device was fabricated by gluing two polarized PVDF films with five pillars by an adhesive (type 810, LEAFTOP, China).

### Physical Characterization and Testing Method

Contact angle (CA) meter (type JC2000D1, POWEREACH, China) was used to characterize the hydrophilicity of the substrate. Fourier-transform infrared (FTIR) (type 6700, NICOLET, US) spectroscopy tests were performed to analyze the composition and phase structure of the samples. Crystallinity was measured by differential scanning calorimeter (DSC) (type DSC 7020, SEICO INST., US). Surface morphologies of samples were characterized by scanning electron microscope (SEM) (type Inspect F50, FEI, US). Electric displacement-electric field (D-E) relations of the poled samples were recorded by ferroelectric analyzer (type HVI40904-523, Radiant, US). Dielectric and dielectric loss constants (ε′ and ε″) were measured by impedance analyzer (type 4294A, Agilent, US).

For pyroelectric measurement, a homemade setup based on electrically modulated method was applied (Additional file 1: Figure S1a). Specifically, square waves at different frequency were produced by a wave generator (type DG1022U, RIGOL Technologies Inc., China). A 980-nm pulsed laser was driven by the square wave and used as a modulated thermal source. The pyroelectric current of the samples was amplified by a homemade current-voltage converting circuit and finally readout by a digital oscilloscope (type DSOX3012A, Agilent, US). For the piezoelectric measurement, a similar setup was built by replacing the laser by a vibrator, which was sinusoidally stimulated through a power amplifier connected with the wave generator (Additional file 1: Figure S1a).

## Results and Discussion

### The PVDF Film

**Fig. 2:**
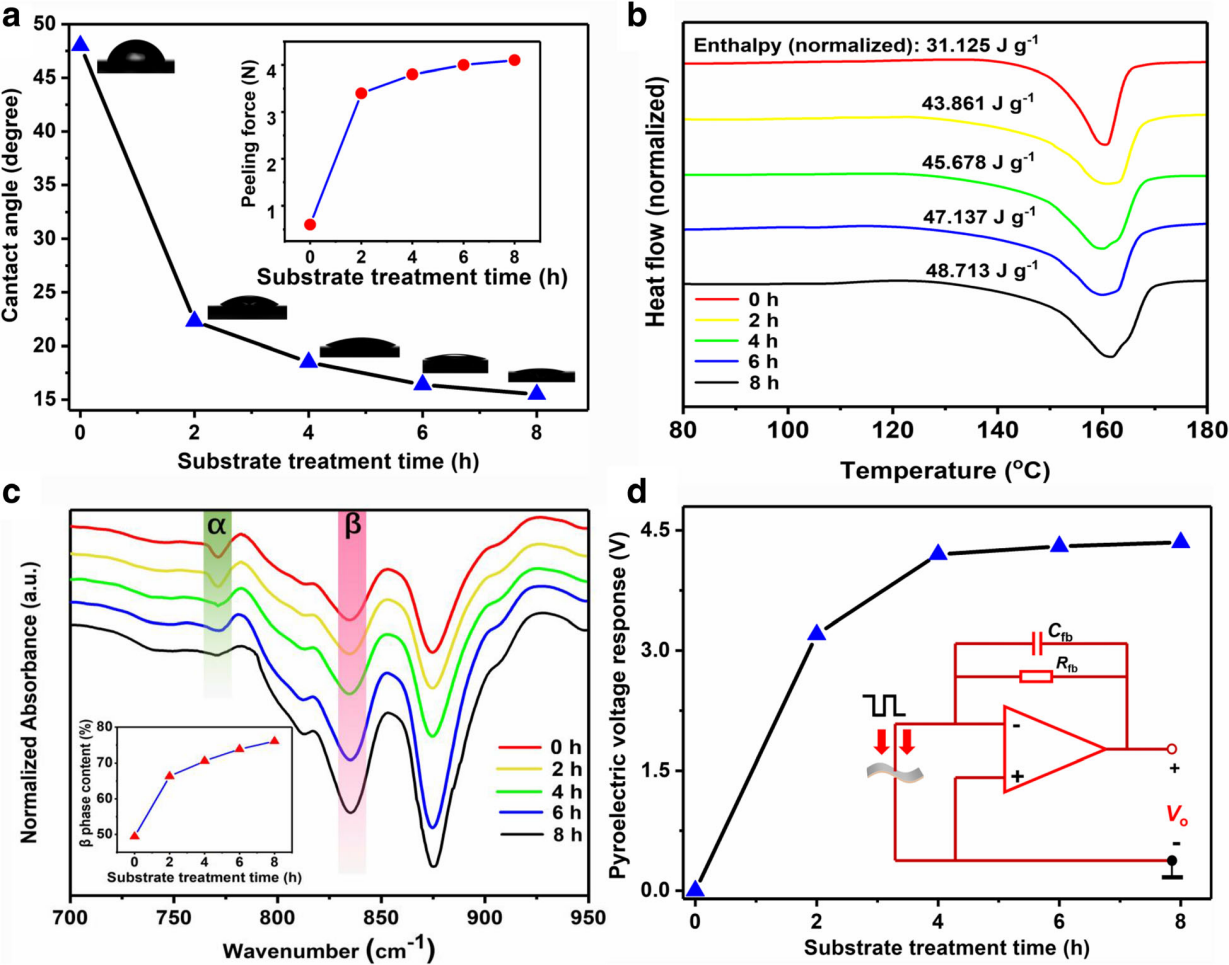
**a** CA of the glass substrates treated in piranha for different time, inset is the peeling force as a function of treatment time. **b** DSC pattern of PVDF samples. **c** FTIR spectra of PVDF samples, inset is β-phase content as a function of treatment time calculated from the FTIR results. **d** Pyroelectric response of PVDF samples without undergoing thermal poling, inset is the simplified schematics of homemade signal readout circuit

Figure 2a shows the CA of the glass substrates immersed in piranha solution for different treatment time. It clearly demonstrates that the hydrophilic property of the substrate is improved after the treatment. The CA continues decreasing with an increasing immersing time and tends to saturate at 8 h. The possible reason can be that more hydrophilic dangling Si-OH groups will generate at the surface of the glass when a longer treatment time is applied. Another proof of this conclusion is the fact that, as the treatment time increases, it became more difficult to peel off the PVDF film from the substrate (the inset of Fig. 2a).

DSC characterizations are carried out to investigate the influence of hydrophilic treatment on crystallinity of PVDF samples. In DSC results, the crystallinity percentage of PVDF can be determined by [40].

1 $$ {X}_{\mathrm{C}}=\left(\frac{\Delta  {H}_{\mathrm{m}}}{\Delta  {H}_{\mathrm{m}}^0}\right)\times 100\%, $$

where *X*_C_ is the crystallinity percentage of PVDF, Δ*H*_m_ is the melting enthalpy of the PVDF, and $\Delta  {H}_{\mathrm{m}}^0$ is the melting enthalpy value of a 100% crystalline PVDF. Figure 2b gives the measured Δ*H*_*m*_ values of PVDF samples casted on substrates with different treatment time. Accordingly, it can be easily calculated that *X*_C_ in the 8-h treated sample increased by larger than 50% compared with the untreated one.

FTIR spectrum is further used to investigate the phase composition in the samples. The peaks at the wavenumber of 764 cm^−1^ and 840 cm^−1^ (Fig. 2c) are usually assigned to characteristics of α and β phase, and the area of the peak (*A*_764_ or *A*_840_) is proportional to the corresponding phase content [41, 42]. As demonstrated in Fig. 2c, A_764_ decreases while *A*_840_ increases monotonically with treatment time. To gain a quantitative sight of the influence of treatment time to phase content in PVDF, the following formula (2) can be applied [42],

2 $$ {F}_{\mathrm{rel}}\left(\upbeta \right)=\frac{X_{\upbeta}}{X_{\upalpha}+{X}_{\upbeta}}=\frac{A_{\upbeta}}{\left({K}_{\upbeta}/{K}_{\upalpha}\right){A}_{\upalpha}+{A}_{\upbeta}} $$

where *X*_α_ and *X*_β_ are the absolute percentage of α and β phases, *A*_α_ and *A*_β_ are peak areas at 764 cm^−1^ and 840 cm^−1^, and K_α_ = 6.1 × 10^4^ cm^2^ mol^−1^ and K_β_ = 7.7 × 10^4^ cm^2^ mol^−1^ are absorptivity constants.

The inset of Fig. 2c shows the calculated β-phase content increases monotonically in a parabolic manner with the treatment time. It reaches a maximum value of 76.05% when treatment time is 8 h, which is about 50% larger than that in the untreated sample. This result, combining with the DSC one, demonstrates that the increased *X*_C_ mainly converts into β phase. We further measure the D-E and ε′ relations of all samples, the results of which also show very similar trends as FTIR (Additional file 1: Figure S2 and S3).

In addition, it is a surprise to find distinct pyroelectric response of PVDF samples on the treated substrates without undergoing further thermal poling procedure (Fig. 2d). The signal conditioning, as indicated by the inset, is realized via a current mode circuit. Similar to the FTIR results, the output signal increases with the treatment time and eventually saturates at 4.3 V when treatment time is 8 h. In comparison, there is no detectable pyroelectric response in the untreated sample (treatment time = 0 h). This result indicates that the hydrophilic groups on the substrate could not only promote the β-phase content, but also be able to polarize the PVDF film. To explore the exact direction of dipole vectors in the sample, a commercially poled PVDF film (Jinzhoukexin, China) with known poling direction is employed as a reference sample. By irradiating two synchronically modulated light sources on both samples, the output signals are recorded with their phases are compared: if two signals are in phase, dipoles in both samples will be parallel to each other; if their phases are reverse, dipoles will be anti-parallel. The results indicate that the direction of dipoles in the treated samples points from the substrate to the film (Additional file 1: Figure S1b and S1c).

**Fig. 3:**
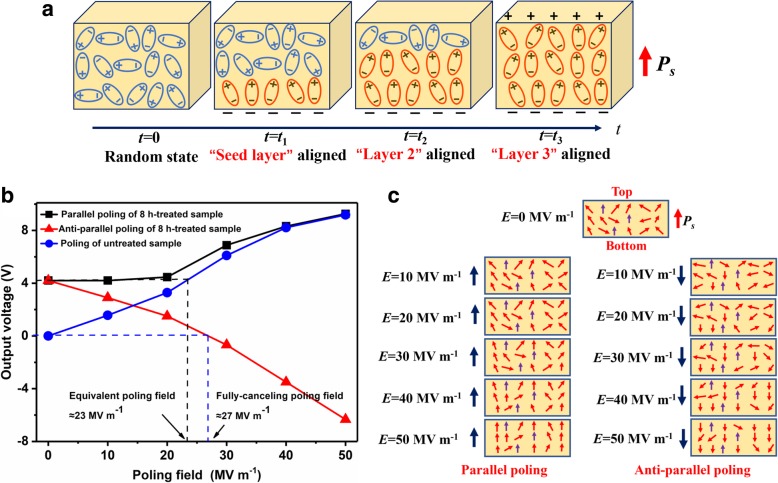
**a** Dipoles alignment relay process in PVDF triggered by the hydrophilic groups among the substrates. **b** Influences of thermal poling field and direction on pyroelectric responses of 8-h treated sample. **c** Schematic diagram of parallel and anti-parallel poling

Based on the above results, the poling mechanism of PVDF films by the hydrophilic groups can be concluded as follows (schematically presented in Fig. 1): The dangling silicon bonds at the surface of the glass substrate will be bonded with the hydroxyl groups after hydrophilic treatment. As the PVDF solution is casted, hydrogen bonds can be formed between fluorine atoms in the VDF units and hydrogen atoms in hydroxyl groups due to their large electric negativity differences. As a result, the dipole vectors in the first sub-nanolayer of PVDF film at the bottom are aligned upwards. This first sub-nanolayer will then play as a seed layer, and afterwards, the adjacent upper sub-nanolayer will be further oriented by electric force, which originates from the already aligned dipole vectors in the seed layer. This process will then repeat in all the above sub-nanolayers as time gets long enough. In other words, the alignment of dipole vectors in PVDF film is relayed bottom-up (Fig. 3a). This dipole alignment relay process may only take place when the molecular chains in PVDF are highly flexible and active before the film is fully cured. Consequently, as the “relay process” finished after the film is fully cured, the β-phase content in the film is promoted and simultaneously the whole film is polarized.

To further investigate the polarization degree in the samples, conventional thermal poling process is performed. Unlike thermal poling of the unpolarized sample, the dipole vectors in the treated samples are already aligned, so that the direction of poling electric field (*E*_p_) should have influence on the film polarization. Therefore, both the parallel and anti-parallel poling is performed. As shown in Fig. 3b, for treated-PVDF poled in the parallel direction, the output signals (*V*_o_) remain stable firstly and then enlarges with the increase of *E*_p_ roughly at the node of 20 MV m^−1^. In comparison, *V*_o_ monotonously increases with the *E*_p_ for the untreated sample in the whole poling range; in addition, *V*_o_ of the untreated sample is always less as *E*_p_ is less than 40 MV m^−1^. As *E*_p_ further increases, *V*_o_ of both samples become equivalent, the maximum of which is 8.8 V as *E*_p_ = 50 MV m^−1^. These results indicate that the polarization value in treated samples as treatment time = 8 h is comparable to the polarization value when it underwent conventional thermal poling at *E*_p_ ≈ 23 MV m^−1^ (equivalent poling field). On the other hand, when the treated sample is poled in reverse, *V*_o_ monotonously decreases with *E*_p_, and, as shown in the figure, *V*_o_ ≈ 0 V as E_p_ ≈ 27 MV m^−1^ (fully-canceling poling field). This phenomenon indicates that the hydrophilically induced polarization can be fully canceled by reverse poling. However, this does not necessarily imply that the induced polarization is fully depolarized; on the contrary, a fraction of the induced polarization still remains as *E*_p_ = 50 MV m^−1^ (Fig. 3c), since the negative maximum *V*_o_ (= − 6.2 V) is obviously less than the maximum *V*_o_ (= 8.8 V) of parallel thermal poled counterparts. These non-depolarizable dipole vectors may need a much larger *E*_p_ (> 50 MV m^−1^) for reorientation, which may be due to their much lower potential energy and higher stability compared with other vectors [43]; this also explains the difference between the equivalent poling field and the fully canceling one.

### The Bilayer Pyroelectric Sensor

**Fig. 4:**
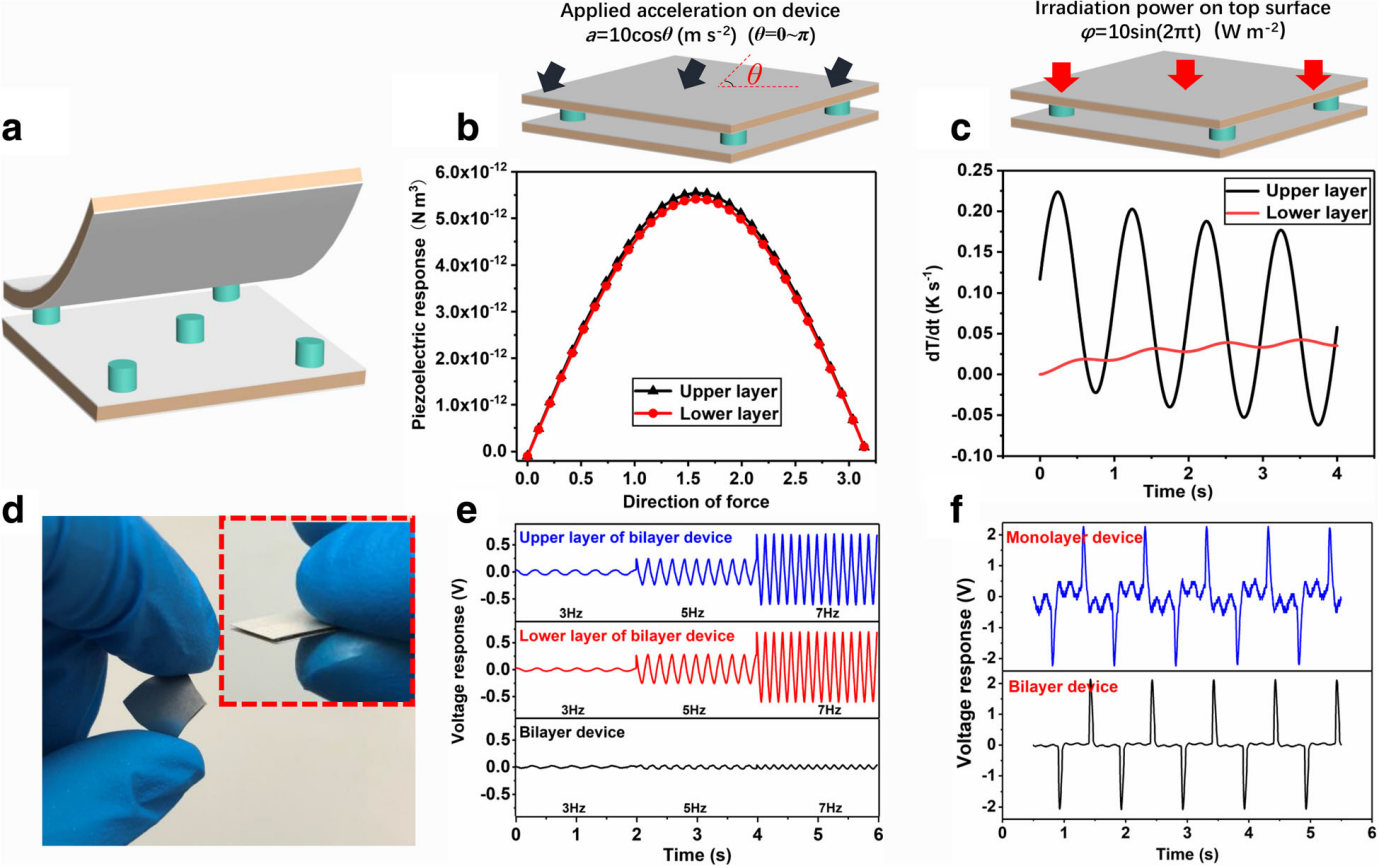
Simulation and measurement results of the bilayer-structured pyroelectric sensor. **a** Explored schematic of the device structure. **b** Model and results of piezoelectric response simulation. **c** Model and results of thermal simulation. **d** Optical photo of the fabricated device. **e** Piezoelectric response at different frequencies. **f** Responses of the bilayer and conventional monolayer devices when simultaneously stimulated by mechanical vibration (5 Hz) and thermal irradiation (1 Hz)

Since all pyroelectric materials inherently possess piezoelectric nature, therefore, an unwanted signal will inevitably be produced when a pyroelectric sensor is mechanically excited through shock or vibration. If two PVDF elements are employed with one as the sensitive material and the other as a reference to compensate the piezoelectric signal, a pyroelectric sensor with minimized piezoelectric noise might be achieved. To this end, we propose a pyroelectric sensor with a novel bilayer structure (Fig. 4a), where two identical PVDF films are mounted together in-between with five tiny pillar separators. In this device, the upper film is the sensitive material and the lower one is the piezoelectric compensator. Two important roles are played by the five pillars: (1) good thermal isolation, i.e., preventing heat dissipation from the upper layer to the lower one; and (2) transferring mechanical vibration between two layers without distortion. Obviously, once the two requirements are fulfilled, a high-quality pyroelectric signal with ultra-low piezoelectric noise can be expected by subtracting the signal of lower element from the upper one.

By using the piezoelectric and thermal models of COMSOL Multiphysics software, mechanical and thermal simulations of the device are conducted to validate the design of this bilayer prototype. As shown in Fig. 4b, piezoelectric responses from both layers show similar variation trends with the exerted acceleration direction. The maximum responses and their differences occur at the normal force direction, where the response of the lower element is about 93.7% of that of the upper one, meaning that the piezoelectric noise of the bilayer sensor can be at least suppressed by 93.7% compared with its monolayer counterpart. Thermal simulation is performed by perpendicularly irradiating a periodic heat wave onto the top of the sensor (Fig. 4c). Compared with the upper layer, temperature change rate (dT/dt) of the lower one nearly keeps stable, indicating heat loss from the upper layer to the lower one is negligible. Consequently, the aforementioned two requirements are indeed satisfied (Note: Fig. 4b and c are simulation results with optimized parameters in Table 1, more details of simulations, i.e., dependencies of piezoelectric and thermal properties of the sensor on geometrical parameters (diameter and height) and position of the pillars, can be found in part 2 of Additional file 1).

**Table 1:** Geometric parameters of the bilayer sensor

Thickness of thin films	Dimensions of thin films	Height of pillars	Diameter of pillars	Distances between two diagonal pillars
50 μm	10 × 10 mm^2^	1 mm	1 mm	11 mm

A bilayer sensor sample is accordingly fabricated (Fig. 4d) based on the 8-h treated samples. As presented in Fig. 4e, obvious piezoelectric-responses of the upper and lower elements are observed, both of which present very similar results at different excitation frequencies. In addition, the variation tendency of response amplitudes with the frequency change turns out to be the typical characteristics of piezoelectric or pyroelectric sensors at low frequency [44]. In comparison, the piezoelectric output of the sample only shows very small signals at all frequencies. Furthermore, the responses of the sample are compared with monolayer one by simultaneously stimulating the samples with a 5-Hz vibration source and a 1-Hz thermal source. The results (Fig. 4f) clearly demonstrate that serious piezoelectric response (about 0.5 V) exists in the signal of the monolayer sample with a pyroelectric signal of 4.4 V, i.e., the signal-to-noise ratio (SNR) = 18 dB, while the bilayer one only has negligible piezoelectric noise (about 0.05 V) with a slightly less pyroelectric signal of 4.1 V, i.e., SNR = 38 dB. These results indicate that the bilayer infrared sensor can be applied in harsh environment where acoustic noise and/or other mechanical noise exist.

## Conclusions

In conclusion, a facile technique for the preparation of pyroelectric PVDF film is developed by casting the precursor on hydrophilic glass substrate. The β-phase content in the prepared sample increases monotonously with the hydrophilic property of the substrate. VDF dipoles in the PVDF film are preferentially aligned in the normal direction, and accordingly, an obvious pyroelectric signal of the sensitive film can be obtained without further undergoing conventional thermal poling. In addition, a novel bilayer pyroelectric sensor is proposed based on the prepared PVDF samples. Compared with conventional monolayer counterpart, piezoelectric noise in the bilayer sensor is suppressed by about 90% while the pyroelectric signal shows nearly no degradation.

## Additional file

Additional file 1: **Figure S1.** (a) schematic of a homemade setup for pyroelectric and piezoelectric measurement; (b) Measurement setup for finding the exact direction of dipole vectors in PVDF test sample; (c) pyroelectric responses of test and reference samples. **Figure S2.** *D-E* curves of PVDF samples at different substrate treatment time. **Figure S3.** ε′(ε″) of PVDF samples (@1 kHz) as a function of substrate treatment time. **Figure S4.** SEM images of the surface morphology of the samples: (a) and (b) are untreated samples; (c) and (d) are 8 h-treated samples. **Table S1.** Material and geometric parameters set values in simulation. **Table S2.** Loads and boundary conditions for simulation. **Figure S5.** Dependences of temperature change rates of both layers on pillars’ height at different pillar’s diameters. **Figure S6.** Dependences of differences between piezoelectric responses of lower and upper layers (*Δ*_piezoelectric_) on pillars’ height at different pillar’s diameters. **Figure S7.** Dependence of piezoelectric response of lower and upper elements on pillars’ position. (DOCX 2935 kb)

## Abbreviations

CA: Contact angle
D-E: Electric displacement-electric field
DSC: Differential scanning calorimeter
FTIR: Fourier-transform infrared
LB: Langmuir-Blodgett
NMP: N-methylpyrrolidone
PDMS: Polydimethylsiloxane
PVDF: Polyvinylidene fluoride
PZT: Lead zirconate titanate
SEM: Scanning electron microscope
SNR: Signal-to-noise ratio
