# Supplementary material for: Self-Polarization of PVDF Film Triggered by Hydrophilic Treatment for Pyroelectric Sensor with Ultra-Low Piezoelectric Noise
Source: Nanoscale Res Lett. 2019 Feb 28;14:72. doi: 10.1186/s11671-019-2906-1 (PMC6395468; doi:10.1186/s11671-019-2906-1)
Supplement: Supplementary file 1 — Figure S1. (a) schematic of a homemade setup for pyroelectric and piezoelectric measurement; (b) Measurement setup for finding the exact direction of dipole vectors in PVDF test sample; (c) pyroelectric responses of test and reference samples. Figure S2. D-E curves of PVDF samples at different substrate treatment time. Figure S3. ε′(ε″) of PVDF samples (@1 kHz) as a function of substrate treatment time. Figure S4. SEM images of the surface morphology of the samples: (a) and (b) are untreated samples; (c) and (d) are 8 h-treated samples. Table S1. Material and geometric parameters set values in simulation. Table S2. Loads and boundary conditions for simulation. Figure S5. Dependences of temperature change rates of both layers on pillars’ height at different pillar’s diameters. Figure S6. Dependences of differences between piezoelectric responses of lower and upper layers (Δpiezoelectric) on pillars’ height at different pillar’s diameters. Figure S7. Dependence of piezoelectric response of lower and upper elements on pillars’ position. (DOCX 2935 kb) [file 11671_2019_2906_MOESM1_ESM.docx]

Electronic Supplementary Information (ESI) for

Self-polarization of PVDF triggered by hydrophilic treatment for pyroelectric sensor with ultra-low piezoelectric noise

Yuming Wu,^a^ Xiaosong Du,^a^ Ruoyao Gao,^a^ Jimeng Li,^a^ Weizhi Li,*^a^ He Yu,^a^ Zhi Jiang,^b^ Zhidong Wang,^c^ Huiling Tai^a^

1. State Key Laboratory of Electronic Thin Films and Integrated Devices, School of Optoelectronic Information, University of Electronic Science and Technology of China, Chengdu 610054, China, E-mail: leewz@uestc.edu.cn;
2. Department of Electrical Engineering and Information Systems, Graduate School of Engineering, The University of Tokyo, Tokyo, Japan；
3. BOE Technology Group Co., Ltd., Chengdu, China;

**Part 1. PVDF film characterization**

**Part 2. Simulation of piezoelectric and pyroelectric responses of bilayer device by changing parameters (height, diameter and position) of the pillars**

**Part 1. PVDF film characterization**

*1. Pyroelectric and piezoelectric measurement setup and methods for finding exact alignment of dipoles in the sample.*


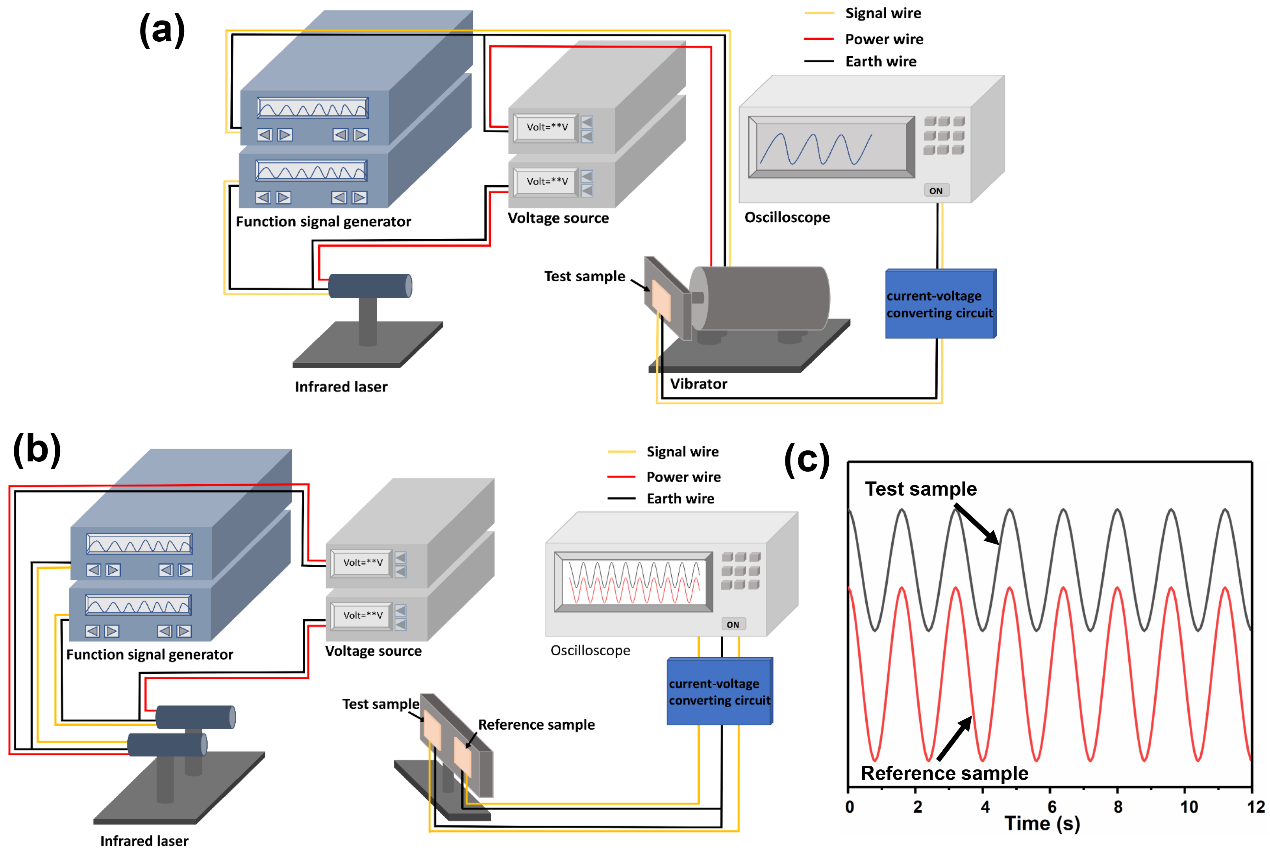


**Additional file 1: Figure S1.** (a) schematic of a homemade setup for pyroelectric and piezoelectric measurement; (b) Measurement setup for finding the exact direction of dipole vectors in PVDF test sample; (c) pyroelectric responses of test and reference samples.


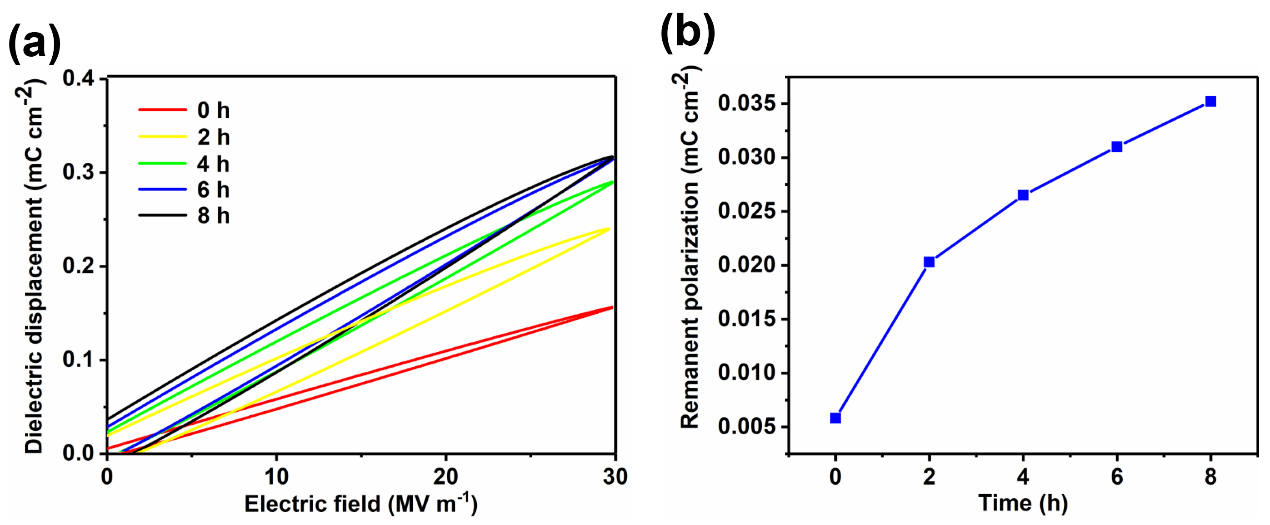
2. *D-E measurements*

**Additional file 1: Figure S2.** *D-E* curves of PVDF samples at different substrate treatment time

To further confirm FTIR results, *D-E* relations of all samples were measured, results of which are presented in Fig. S2a. It obviously demonstrates that *D-E* relations are significantly influenced by substrate treatment time. By extracting remnant polarization (*P*_r_) data in the figure, *P*_r_-treatment time relation for all composites are given in Fig. S2b. It shows that the relation is similar to β phase content-treatment time relation in Fig. 2c, which is well confirming FTIR results.

3. *Dielectric measurements of samples*





**Additional file 1: Figure S3.** ε’(ε’’) of PVDF samples (@1kHz) as a function of substrate treatment time

For dielectric materials the following Eq. S1 holds:

$P=\frac{\sum m}{\Delta V}=\varepsilon_{0}\chi_{e}E=\varepsilon_{0}(\left|\varepsilon_{r}\right|-1)E$ (S1)

where *P*, *χ_e_*, *ε_r_* are respectively the polarization density, the susceptibility and permittivity of the dielectric. *ΔV* is a small volume element in the dielectric and Σ*m* is the sum of molecular electric moments (dipole moments) in Δ*V*. *E* is the electric field, *ε_r_* is the permittivity of the vacuum and can be calculated by Eq. S2:

$\varepsilon_{r}=\varepsilon^{'}+j\varepsilon^{''}$ (S2)

where *j* is the imaginary unit, *ε*” and *ε*’ are dielectric constant and loss, respectively. Since *ε*”<< *ε*’, Eq. S2 can be reduced to

$P=\frac{\sum m}{\Delta V}\approx\varepsilon_{0}(\varepsilon^{'}-1)E$ (S3)

On the other hand, Σ*m* in equation S2 depends on either magnitude of molecular dipole moment (*M*_d_) or/and its orientational ability along applied electric filed. For PVDF, *M*_d_ can be neglected in α or γ phases, thus results in Fig. S3 clearly indicate β (or δ) phase content increases or/and VDF dipoles are more easily aligned as the substrate treatment time increases.

4. *SEM photos of samples*


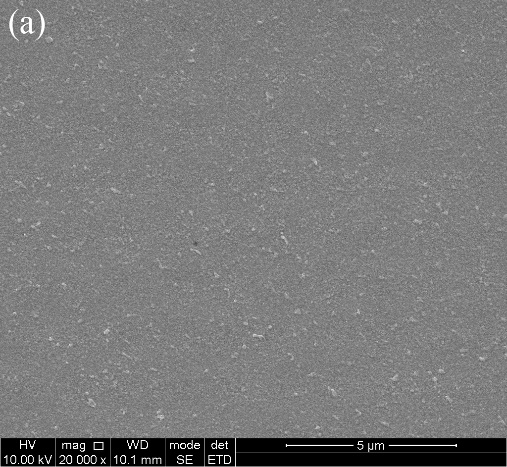

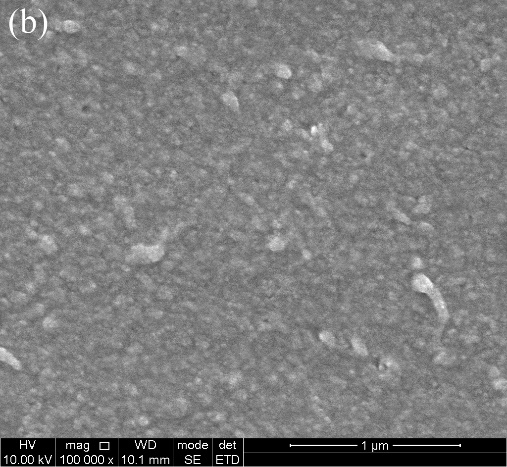

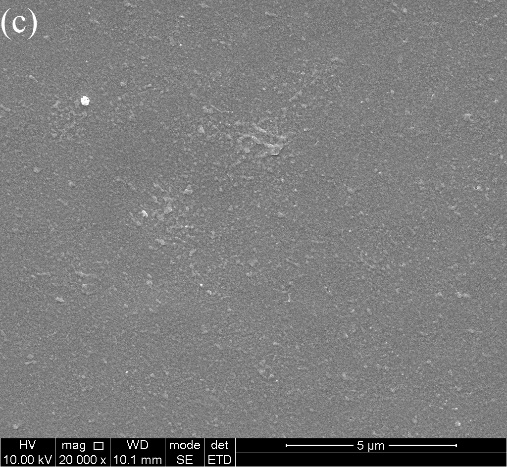

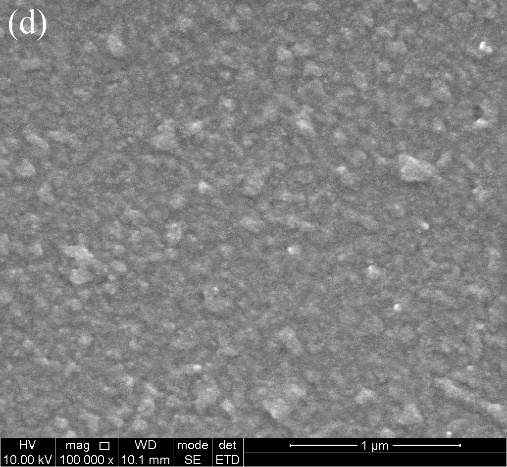


**Additional file 1: Figure S4.** SEM images of the surface morphology of the samples, which indicates that piranha treatment to the substrates has no obvious influence on the surface morphology of PVDF samples: (a) and (b) are untreated samples; (c) and (d) are 8 h-treated samples.

**Part 2. Simulation of piezoelectric and pyroelectric responses of bilayer device with variation of parameters (height, diameter and position) of the pillars**

**Table S1** Material and geometric parameters set values in simulation

| Material parameters | | | | Geometric parameter | | | |
| --- | --- | --- | --- | --- | --- | --- | --- |
| / | Density (kg m^-3^) | Thermal conductivity (W m^-1^K^-1^)$W/(m\cdot K))$ | Heat capacity  (J kg^-1^K^-1^) | Diameter  （mm） | Thickness/Heght（μm） | Length (mm) | Width (mm) |
| PVDF | 1780 | 0.15 | 1348 | - | 50 | 10 | 10 |
| PDMS(pillars) | 970 | 0.16 | 1460 | 1~3 | 100~1200 | - | - |

**Table S2** Loads and boundary conditions for simulation

|  | Loads | Boundary conditions |
| --- | --- | --- |
| Piezoelectric simulation | Applied acceleration on top surface  *a*=10cos*θ* (m s^-2^) (*θ=*0*~π*) | - |
| Pyroelectric simulation | Irradiation power on top surface  *φ*=10sin(2πt)（W m^-2^） | Temperature of bottom surface of the lower layer=293.15K |

*1. Pyroelectric response of lower and upper layers vs. pillars’ height (H_p_) and diameter (D_p_)*


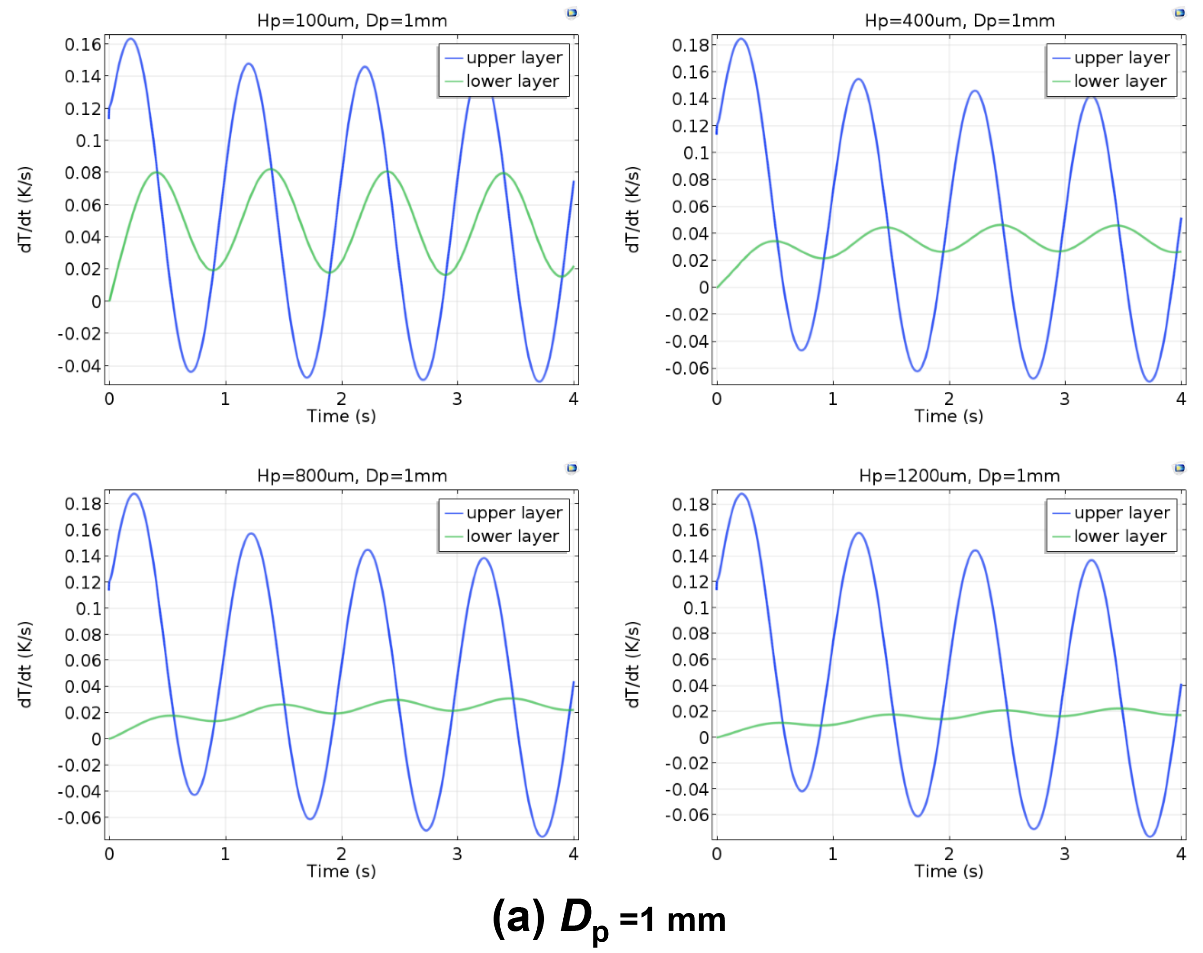


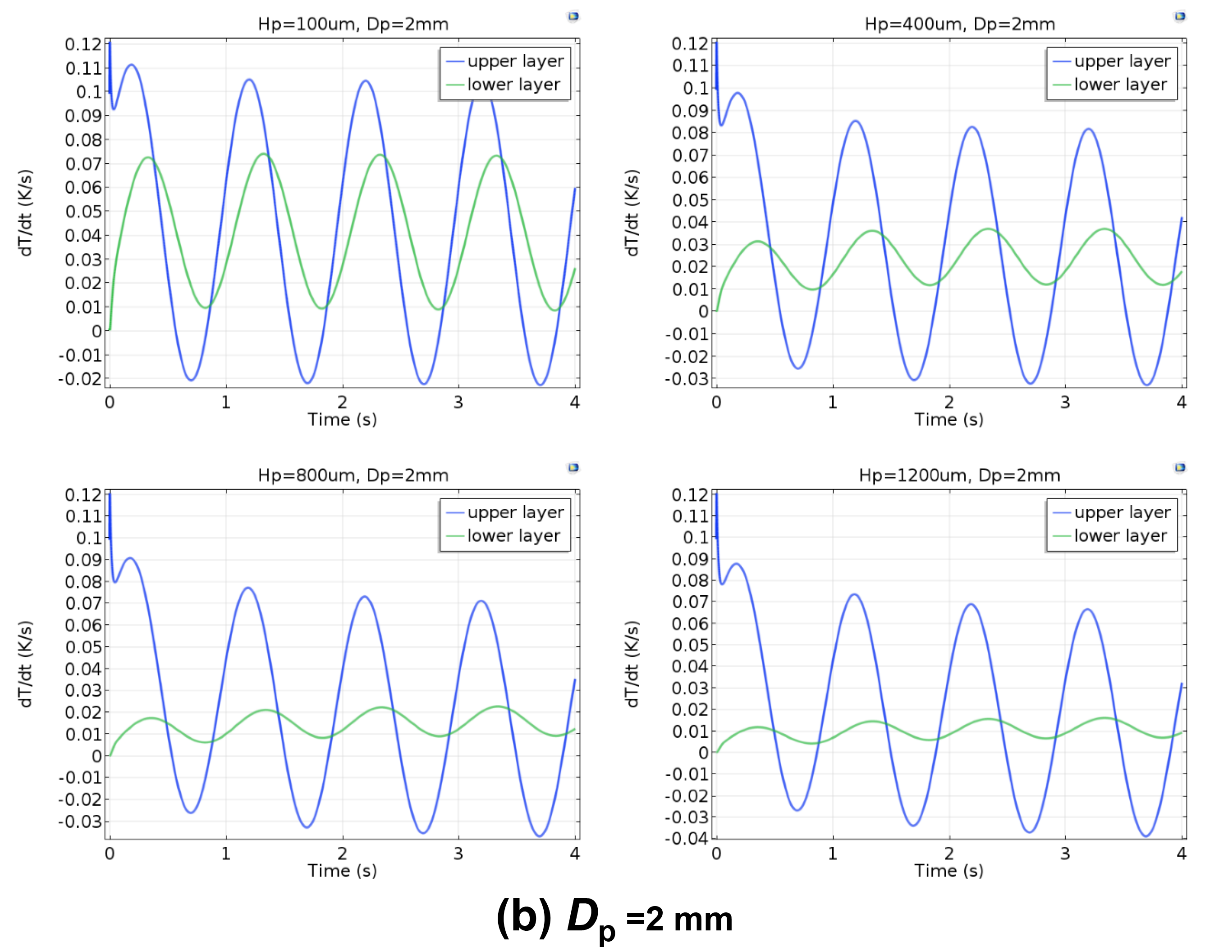


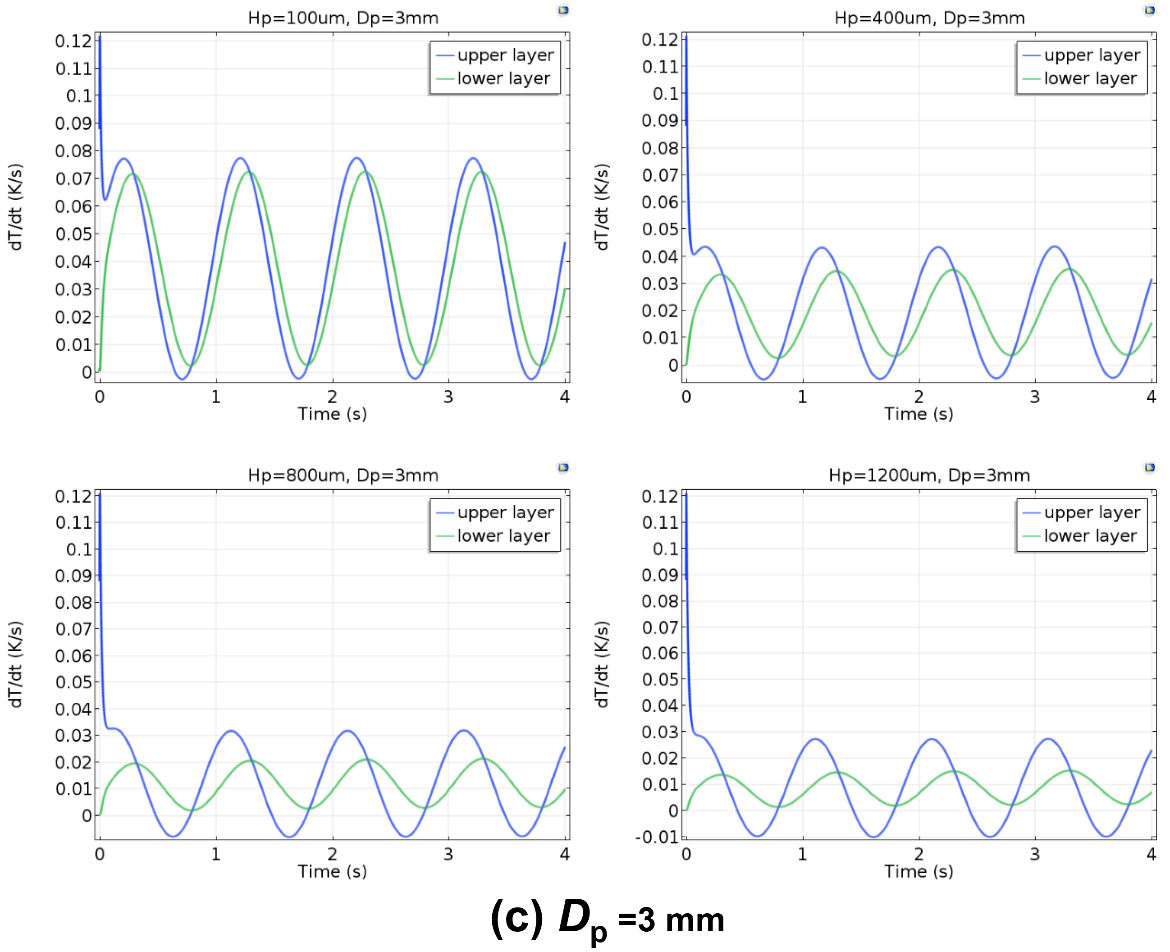


**Additional file 1: Figure S5.** Dependences of temperature change rates of both layers on pillars’ height at different pillar’s diameters

As shown in Fig. S5a, b and c, both the increase of pillars’ height and decrease of pillar’s diameter are beneficial for the thermal insulation between two layers; as pillars’ diameter is larger than 1mm, thermal loss from the upper layer to the bottom one is not negligible whatever the height is. Results in Fig. S5a indicates that as the *D*_p_ = *H*_p_ =1mm, thermal loss of the upper layer is less than about 5%.

*2. Piezoelectric response of lower and upper elements vs. pillars’ height and diameter*


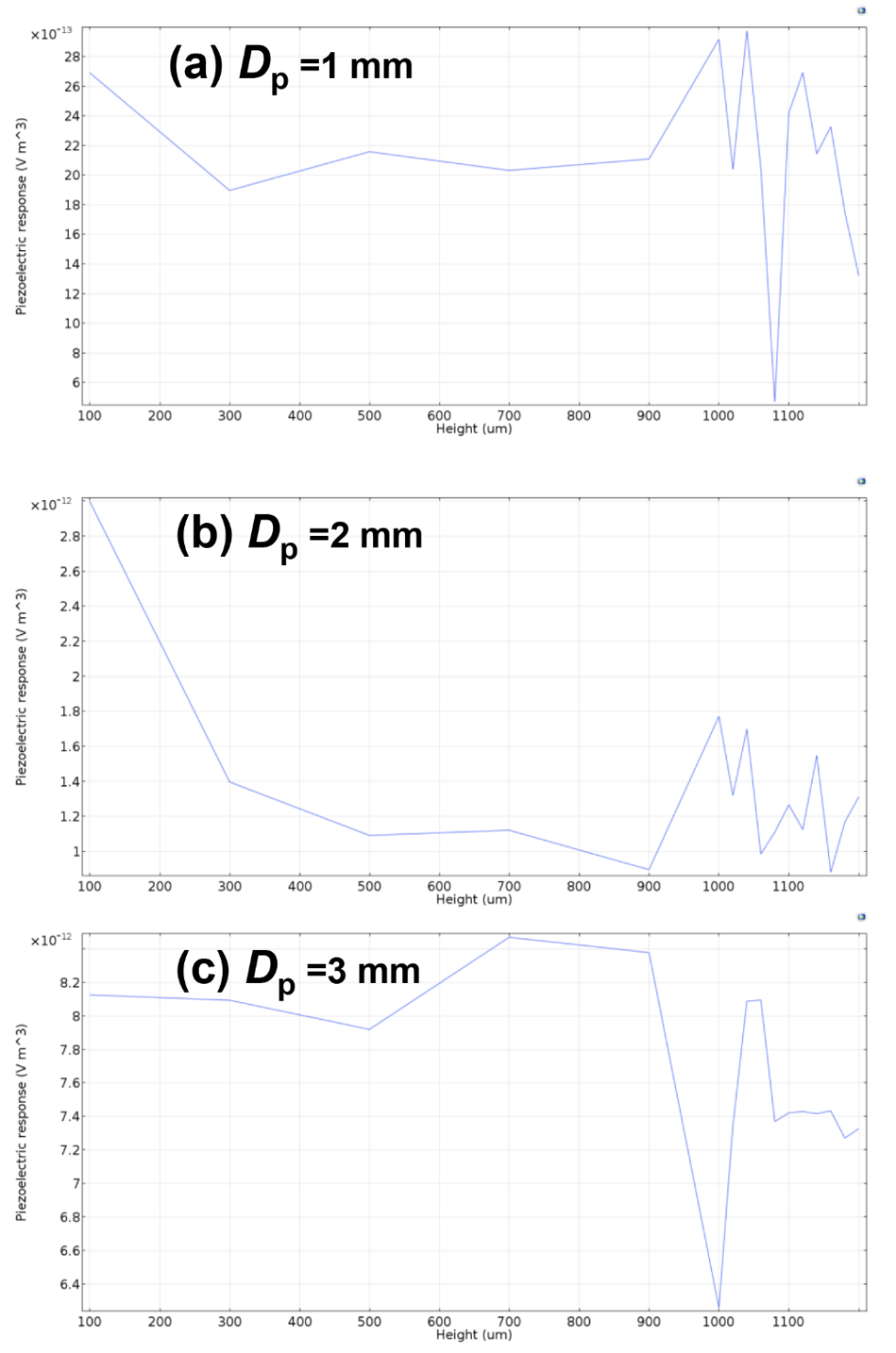


**Additional file 1: Figure S6.** Dependences of differences between piezoelectric responses of lower and upper layers (*Δ*_piezoelectric_) on pillars’ height at different pillar’s diameters

Since the largest *Δ*_piezoelectric_ appears when the exerted constant force is in the normal direction (θ=π/2) by varying the force direction (0~π, see Fig.4 in the manuscript), piezoelectric simulations were conducted by fixing θ at π/2. As demonstrated in Fig. S6, *Δ*_piezoelectric_ increases with the diameter of the pillars. Especially, *Δ*_piezoelectric_ increases by a few times as *D*_p_ increases to 3 mm from Fig. S6a to c, this probably attributes to the large stiffness as the pillars get thick enough, which therefore distorts strain transfer between the two layers. On the other hand, *Δ*_piezoelectric_ fluctuates irregularly as *H*_p_ gets larger, as shown in Fig. S6a, the minimum *Δ*_piezoelectric_ occurs as *H*_p_≈1080 μm. Based on results in Fig. S5 and S6, *H*_p_=*D*_p_=1 mm is therefore chosen.

*3. Piezoelectric response of lower and upper elements vs. pillars’ position (d).*
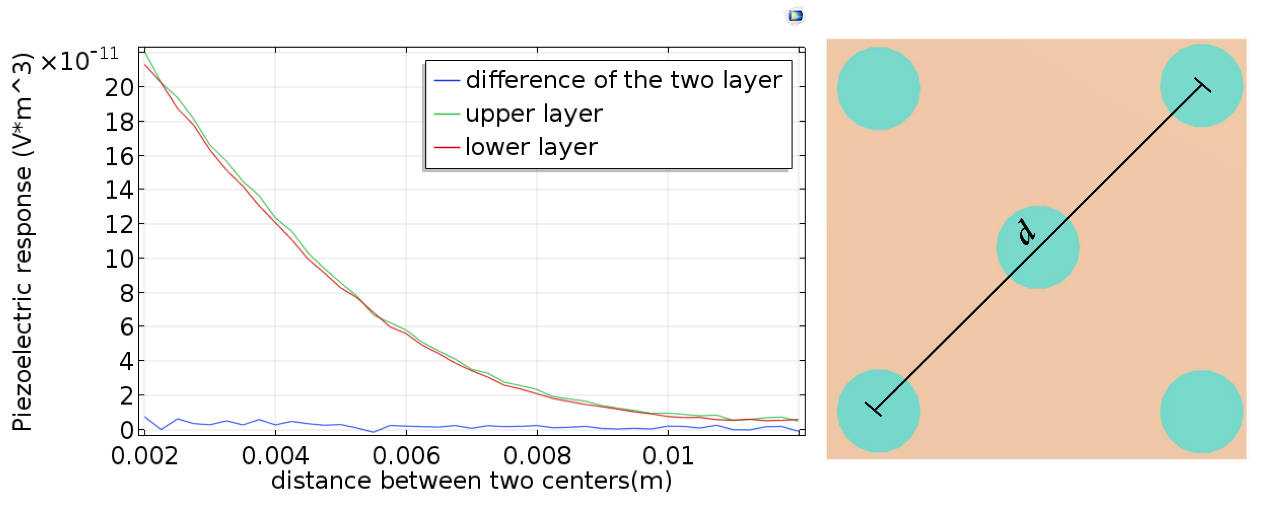


**Additional file 1: Figure S7.** Dependence of piezoelectric response of lower and upper elements on pillars’ position

We further investigated the influences of 4 surrounding pillars’ positions, i.e., distances between two diagonal pillars (d), on piezoelectric response of device by fixing *H*_p_ and *D*_p_ at 1 mm. As indicated in Fig. S7, while *d* indeed has significant impact on piezoelectric responses of both elements (which monotonously decrease with *d*), *Δ*_piezoelectric_ nearly remains unchangeably small. Comparatively, *Δ*_piezoelectric_ reaches its minimum as *d*=11 mm.

Based on above results, optimal parameters of the pillars applied for real device fabrication are *H*_p_=*D*_p_=1 mm and *d*=11 mm.
